# 

**Published:** 1843-10

**Authors:** 


### CONTENTS

OF NO. IV.

### ORIGINAL COMMUNICATIONS.

ESSAYS AND CASES.

Art. I.—Contributions to Morbid Anatomy. By Bennet Dowler, M.D., of New Orleans. - - - - - 241
Art. II.—A New Method of removing Foreign Bodies from the Joints. By G. Goyrand, M.D., Chief Surgeon to the Hospital at Aix; Corresponding member of the Royal Academy of Medecine, &c. Translated from the French for the Western Journal of Medicine and Surgery, by E. L. Grant, M.D., of Louisville. - 266
Art. III.—Cases of Influenza, with Remarks. By Solon Borland, M.D., of Louisville. - - - - - 274

SELECTIONS FROM AMERICAN AND FOREIGN JOURNALS.

Medical Faculty of Minorca. - - - - - 290
Necrosis of the Skull. - - - - - - 296
Extraordinary Case, in which the Arm was torn off at the Shoulder-joint, by machinery. - . - - 299
Emmenagogue Medicines. - - - - - - 300
The Free-and-Easy Style. - - - - - - 301

An account of a case in which a Foreign Body was lodged in the Right Bronchus. - - - - - 303
Rosas's Operation for Cataract. - - - - 304
Researches on the Frequency of Cancer. - - - 306
Black Cataract. - - - - - - 310
New Limits for the period of Lactation. - - - 311
Diagnostic Sign of Dislocation of the Thigh-Bone into the Ischiatic Notch. - - - - - 311
Quinine in Traumatic Fever. - - - - 312
Wenzel the Oculist. - - - - - - 313
Moveable Kidney. - - - - - - 313
On Lateral Curvature of the Spine. - - - - 314
Van Arsdale on Obstetrical Auscultation. - - - 317

ORIGINAL INTELLIGENCE.

Health of the South. - - - - - 319
Pennsylvania Medical College, Philadelphia. - - 319
Smith's Minor Surgery. - - - - 320

